# Short-term exposure to CO and NO_2_ and Kawasaki disease onset in a cold region of China: a distributed lag non-linear time-series study

**DOI:** 10.3389/fpubh.2026.1853103

**Published:** 2026-06-17

**Authors:** Jingchun Yang, Yishan Sun, Yan Zhao, Yuzhu Chen, Hongxin Xu, Jiaqing Zhang, Tao Song, Xuguang Zhang, Zhiqiang Li

**Affiliations:** 1 Department of Pediatric Surgery, the Sixth Affiliated Hospital of Harbin Medical University, Harbin, China; 2 Department of Nutrition and Food Hygiene, The National Key Discipline, School of Public Health, Harbin Medical University, Harbin, China; 3 Tianjin Children’s Hospital, Tianjin, China; 4 Department of Cardiology, the First Affiliated Hospital of Harbin Medical University, Harbin, China; 5 Sydney Smart Technology College, Northeastern University, Qinhuangdao, China; 6 Department of Child Healthcare, Heilongjiang Hospital of Beijing Children's Hospital, Harbin, China; 7 Department of Cardiac Surgery, National Children's Medical Center, Beijing Children's Hospital, Capital Medical University, Beijing, China

**Keywords:** air pollutants, carbon monoxide, cold regions, distributed lag model, Kawasaki disease, nitrogen dioxide

## Abstract

**Background:**

Short-term exposure to ambient air pollution has been associated with Kawasaki disease (KD), however, evidence from cold climates remains limited. We aim to evaluate the association between short-term ambient pollutant exposure and KD onset in a cold region of China.

**Methods:**

We conducted an ecological time-series study based on aggregated daily counts of hospital-diagnosed KD onset cases. Individual clinical records from 2,562 hospitalized patients diagnosed with KD at two large tertiary medical centers between January 1, 2015, and December 31, 2022, were used to identify eligible cases and determine the dates of first fever onset. Our analysis included a year-round evaluation of multiple ambient pollutants pertinent to both heating and non-heating periods typical of cold regions. We assessed six pollutants (PM_2.5_, PM_10_, SO₂, NO₂, CO, and O₃) using a generalized linear model with distributed lag nonlinear models (GLM–DLNM), and performed stratified analyses of CO and NO₂ by gender and heating season.

**Results:**

High CO concentration (RR = 1.172; 95% CI: 1.024–1.340) and high NO₂ concentration (RR = 1.117; 95% CI: 1.009–1.237) were associated with elevated KD risk at lag 0 under single-day lag analysis. Significant cumulative effects were observed for CO (lag 0–6: RR 1.838; 95% CI: 1.022–3.306) and NO_2_ (lag 0–2: RR 1.298; 95% CI: 1.003–1.680). Cumulative exposure–response curves indicated that KD risk increased at concentrations above approximately 1.85 mg/m^3^ for CO and 53.6 μg/m^3^ for NO₂. In stratified analyses, these associations appeared more evident among females and during the heating season. Furthermore, O_3_, SO_2_, PM_2.5_ and PM_10_ showed no significant links.

**Conclusion:**

Short-term exposure to CO and NO₂ was associated with increased daily hospital-diagnosed KD onset cases. Reducing CO and NO_2_ may have potential children health relevance, although further studies are needed to confirm these associations and clarify underlying mechanisms.

## 1 Introduction

Kawasaki disease (KD) is an acute, self-limiting systemic vasculitis of unknown etiology that predominantly affects infants and young children, with more than 80% of cases occurring in those under five years of age (1). It is characterized by systemic inflammation of small- to medium-sized arteries, with typical clinical manifestations including prolonged fever, bilateral non-exudative conjunctival injection, polymorphous rash, erythematous changes of the lips and oral mucosa, and cervical lymphadenopathy (2). Without timely treatment, KD can lead to severe cardiac complications, such as coronary artery dilatation and aneurysms. These complications often result in long-term cardiovascular sequelae, making KD a leading cause of acquired heart disease in children worldwide (3). In recent years, KD incidence has risen markedly in the Western Pacific region, especially in Japan and South Korea, where rates exceed 200 per 100,000 person-years among children under five. Beijing and Taiwan also report substantial KD burdens, with annual incidence rates above 50 per 100,000 person-years (4). KD can occur in all seasons, but the timing of peak incidence varies across regions (5). In China, cities such as Shanghai and Beijing experience the highest incidence from May to August, while Taiwan reports a peak in July. Japan and South Korea show increased case numbers in both January and July to September, whereas the United States and the United Kingdom typically observe peak incidence during February and March. Although previous studies have implicated bacterial, viral, and fungal infections maybe contribute to KD onset (6, 7), its precise etiology remains unclear.

Recent studies have established a direct link between exposure to air pollution and the onset of KD, and have shown a significant association with an increased risk of cardiovascular diseases (8–10). For example, a study from Japan that included 224 KD cases found a positive relationship between KD incidence and elevated NO concentrations at a 1-day lag, as well as SO₂ concentrations at a 0-day lag (11). Similarly, a large-scale retrospective study in South Korea (*n* = 51,486) reported that high levels of PM_2.5_ and SO_2_ significantly increased the risk of KD onset (12). Notably, KD incidence is higher during the colder months in Japan, South Korea, and China. In high-latitude cold regions, combustion-related pollutants such as CO and NO₂ typically increase markedly during the heating season. This seasonal variation drives unique exposure patterns in cold regions differ from those in other studied areas, which may lead to distinct effects on KD onset. Therefore, research focused on cold regions is crucial, as different climatic conditions and increased pollution exposure may heighten the risk of KD.

To address the association between air pollutant exposure and KD onset in cold regions, this study conducted the first ecological time-series study based on 2,562 cases of pediatric KD hospitalizations in Heilongjiang Province, China, aiming to clarify the relationship in children living in cold climates. These findings may contribute to a greater understanding of potential environmental factors associated with KD onset in cold regions and provide evidence for future environmental health studies.

## 2 Materials and methods

### 2.1 Study area

Heilongjiang Province (centered approximately at 125°E, 46°N) is located in Northeast China and is the northernmost provincial region of the country. In 2022, it had a total population of about 31.9 million residents (13). The province has a continental monsoon climate with long and extremely cold winters. The annual mean temperature ranges from −4 °C to 5 °C, and winter temperatures frequently fall below −20 °C, with nighttime temperatures occasionally dropping below −30 °C. Due to these climatic conditions, centralized coal-fired heating systems are generally in operation from October 20 to April 20 each year (14). Coal combustion and other heating-related emissions during this period may contribute to elevated ambient pollutant concentrations.

### 2.2 Study design and participants

This study was approved by the Ethics Committee of the Sixth Affiliated Hospital of Harbin Medical University (Approval No. LC2024-133), with permission to use relevant clinical data from the electronic medical record system for research analysis. Medical records of children discharged with a diagnosis of KD between January 1, 2015, and December 31, 2022, were collected from the First Affiliated Hospital of Harbin Medical University and Heilongjiang Hospital, Beijing Children’s Hospital. The collected information included age, sex, date of fever onset, and residential address. KD was diagnosed according to the American Heart Association clinical criteria (2). Complete KD was defined as fever lasting ≥5 days with at least four of the five principal clinical features, including bilateral non-exudative conjunctival injection, oral mucosal changes, polymorphous rash, extremity changes, and cervical lymphadenopathy. All diagnoses were confirmed by two experienced pediatricians according to standard clinical diagnostic criteria. Cases with residential addresses outside Heilongjiang Province or with unclear fever onset dates were excluded, and readmissions within 14 days were considered part of the same disease episode. The primary outcome of this study was the daily number of KD onset cases. Informed consent was not required because anonymized electronic clinical data were used.

### 2.3 Environmental exposure data sources

Concentrations of particulate matter (PM_10_ and PM_2.5_), ozone (O_3_), nitrogen dioxide (NO_2_), sulfur dioxide (SO_2_), and carbon monoxide (CO) were retrieved from the China National Environmental Monitoring Center. These pollutants were measured by the national ambient air quality monitoring network using standardized continuous automatic monitoring methods and quality-control procedures. Briefly, PM_10_ and PM_2.5_ were monitored using continuous particulate matter analyzers, while SO_2_, NO_2_, O_3_, and CO were measured using continuous automated gas-monitoring systems according to national technical specifications. Pollutant data were obtained from 31 environmental monitoring stations across Heilongjiang Province, including 2 baseline stations, 10 basic stations, and 19 general stations. Given its pronounced diurnal variation and photochemical formation during daylight hours, O_3_ was summarized as the daily maximum 8-h moving average (O_3_-8h) to better reflect health-relevant short-term ozone exposure (15). In contrast, PM₂.₅, PM₁₀, SO₂, NO₂, and CO were summarized as daily 24-h mean concentrations, which are commonly used exposure metrics in short-term time-series studies. Meteorological data were obtained from the Heilongjiang Provincial Meteorological Bureau and included daily mean temperature (Temp, °C) and relative humidity (RH, %). Daily missing data rates for both air-pollutant and meteorological variables were <0.05%. To preserve the continuity of the daily time-series data, missing values were handled using multiple imputation. The imputation model included air pollutants and meteorological variables, including PM₂.₅, PM₁₀, SO₂, NO₂, CO, O₃-8 h, daily mean temperature, and relative humidity. Imputed datasets were generated, and the imputed values were used to construct the complete daily dataset for the main analyses.

### 2.4 Subgroup analysis

To investigate potential effect modification in the association between air pollutants and KD onset, we performed stratified analyses by sex and heating period (from October 20 to April 20 of the following year). This classification was based on the fact that pollutant levels in high-latitude cold regions exhibit pronounced seasonal contrasts, primarily driven by centralized heating emissions, resulting in markedly different exposure profiles across the year. For each subgroup, we estimated single-lag and cumulative lag effects using the same modeling framework as the main analysis, allowing direct comparison of exposure–response differences across sex and seasonal strata.

### 2.5 Statistical analysis

We assessed the association between ambient pollutant concentrations and the daily number of KD onset cases using a quasi-Poisson generalized linear model with a log link, combined with a distributed lag non-linear model (GLM-DLNM), to account for potential overdispersion in the daily count data. The maximum lag was set to 30 days (lag = 30) to capture delayed associations between pollutant exposure and KD onset (16). DLNM can simultaneously model non-linear exposure–response relationships and delayed exposure effects through the specification of a cross-basis function (17). For each pollutant, the cross-basis function was specified using a natural cubic spline with 3 degrees of freedom (df) for the exposure–response dimension and a natural cubic spline with 4 df for the lag dimension. The df values for the spline functions were selected based on the quasi-Akaike Information Criterion (QAIC). Long-term and seasonal patterns were controlled by including a smooth function of calendar time with 7 df per year (18). Daily mean temperature and relative humidity were included as meteorological confounders and modeled using natural cubic splines with 6 df and 3 df, respectively (19). Day of the week (DOW) and public holidays were additionally included as categorical variables to account for weekly and holiday-related variations.

The model was specified as:


$$\begin{array}{l} \log[E(Y_{t})]=\alpha +\beta X_{t}+\mathrm{ns}(\text{Time},7\times \text{Years})+\mathrm{ns}(\mathrm{MT},6)\\ +\mathrm{ns}(\mathrm{RH},3)+\mathrm{DOW}+\text{Holiday} \end{array}$$

In the formula: where *Y_t_* is the daily number of Kawasaki disease (KD) onsets on day *t*; *α* is the intercept term; *β* is the regression coefficient associated with air pollution exposure; *X_t_* denotes the cross-basis matrix for air pollutant concentration and lagged days; *ns(·)* represents a natural cubic spline function; *Time* is the time variable used to control long-term and seasonal trends with 7 × Years degrees of freedom; *MT* is the daily mean temperature (°C) modeled with 6 degrees of freedom; *RH* is the daily relative humidity (%) modeled with 3 degrees of freedom; *DOW* is a categorical variable representing the day of the week; and *Holiday* is a categorical variable representing public holidays. All associations are presented as Relative Risks (RR) with corresponding 95% Confidence Intervals (95% CI).

Predicted exposure–lag–response associations were obtained using the crosspred() function from the dlnm package. Single-lag effects, cumulative lag effects, exposure–response curves, and three-dimensional exposure–lag surfaces were generated for visualization. For descriptive purposes, we identified the approximate concentrations at which the lower 95% confidence bounds of the model-predicted cumulative RRs crossed 1 using linear interpolation. These values were used to describe the upper concentration ranges where the estimated cumulative associations became statistically elevated. Relative risks were estimated for specified exposure contrasts relative to the reference concentration, rather than per one-unit increase in pollutant concentration. The median concentration was used as the reference value, and the upper-tail percentile concentrations were used to characterize high-exposure contrasts.

Several sensitivity analyses were conducted to evaluate the robustness of the results. First, two-pollutant models were fitted after examining pairwise correlations between pollutants, and pollutant pairs with an absolute correlation coefficient |r| > 0.7 were not included simultaneously in the same model (Supplementary Table S1) (20, 21). Second, we varied the degrees of freedom for the smooth functions of time, temperature, and relative humidity (time: 6–8 df per year; temperature: 4–6 df; humidity: 3–5 df) to assess model stability. In addition, sensitivity analyses were conducted using more conventional exposure contrasts, including the 75th versus 50th percentile and the 90th versus 50th percentile.

All statistical analyses were performed using R (version 4.4.1; R Foundation for Statistical Computing, Vienna, Austria). Data preprocessing and management were conducted with the “dplyr” package, and distributed lag non-linear models were fitted using the “dlnm” and “survival” packages. Graphical presentations were produced using “ggplot2”, and spline functions were implemented with the “splines” package. All statistical tests were two sided, and *p* values < 0.05 were considered statistically significant.

## 3 Results

### 3.1 Baseline characteristics and exposure profiles

**Figure 1:**
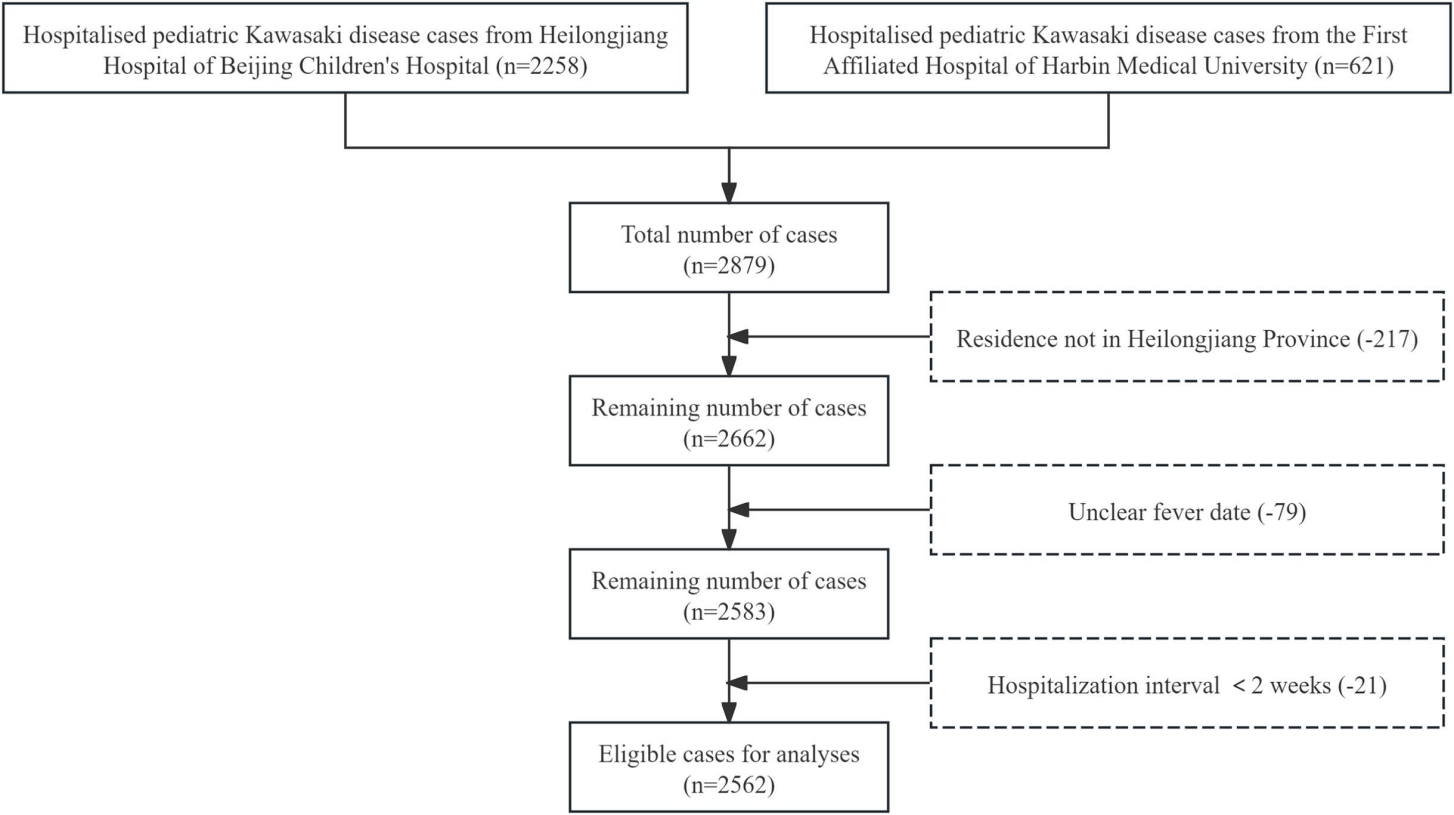
Study population and the exclusion criteria.

During the study period, a total of 2,879 hospitalized cases of KD were recorded. Among them, 2,562 eligible cases were retained for analysis (Figure 1). The characteristics of aggregated daily counts, along with ambient air pollutant concentrations and meteorological indicators, are shown in Table 1. Male patients accounted for the majority of cases (*n* = 1,546), with a male-to-female ratio of 1.52:1. In contrast to previous epidemiological findings that KD tends to peak in summer (22, 23), our results from this cold-climate region showed a nearly equivalent number of cases between the heating season (*n* = 1,273) and the non-heating season (*n* = 1,289).

**Table 1:** Descriptive statistics of air pollutants, meteorological factors and KD during the study period.

Variables	Mean ± SD*^d^*	Min.*^e^*	P25*^a^*	P50*^b^*	P75*^c^*	Max.*^f^*
Air pollutants						
PM_2.5_ (μg/m^3^)	33.05 (29.00)	5.70	15.23	23.89	40.96	326.47
PM_10_ (μg/m^3^)	21.71 (16.26)	4.26	13.37	18.03	25.01	250.63
SO_2_ (μg/m^3^)	13.88 (10.65)	4.48	7.04	9.73	16.97	89.55
NO_2_ (μg/m^3^)	22.66 (9.58)	6.86	15.91	20.47	27.45	73.19
O_3_-8h (μg/m^3^)	54.20 (18.08)	10.63	39.88	51.90	66.32	142.71
CO (mg/m^3^)	0.66 (0.27)	0.30	0.48	0.58	0.76	2.69
Meteorological parameters						
Temperature (°C)	4.49 (14.76)	−28.61	−9.69	6.55	17.99	27.82
Relative humidity (%)	62.97 (14.32)	21.06	53.27	64.15	73.74	94.65
Kawasaki disease						
Total (*n* = 2,562)	0.88 (0.97)	0.00	0.00	1.00	1.00	6.00
Male (*n* = 1,546)	0.53 (0.75)	0.00	0.00	0.00	1.00	5.00
Female (*n* = 1,016)	0.35 (0.60)	0.00	0.00	0.00	1.00	4.00
Heating (*n* = 1,273)	0.87 (0.98)	0.00	0.00	1.00	1.00	5.00
Non-heating (*n* = 1,289)	0.89 (0.96)	0.00	0.00	1.00	1.00	6.00

a, P25 denotes the 25th percentile; b, P50 denotes the 50th percentile; c, P75 denotes the 75th percentile; d, SD refers to standard deviation; e, Min stands for minimum; f, Max stands for maximum.

**Figure 2:**
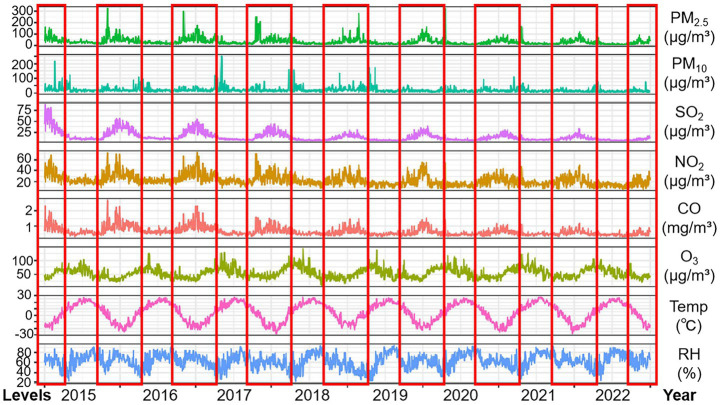
Annual time series of ambient pollutants and meteorological factors in Heilongjiang during 2015 to 2022. The red rectangular frames denote heating periods.

To better illustrate seasonal fluctuations in environmental exposures, we plotted the time-series distributions of major air pollutants and meteorological variables during the study period (Figure 2). Across all years, PM₂.₅, PM₁₀, SO₂, NO₂, and CO consistently demonstrated recurrent wintertime surges, forming pronounced peaks within each heating-season interval. These patterns reflect substantial pollutant accumulation driven by increased combustion and reduced atmospheric dispersion in cold months. In contrast, O₃ exhibited an opposite seasonal pattern, with clear summertime elevations likely related to stronger solar radiation and higher temperatures, which promote photochemical reactions involving ozone precursors.

Overall, the seasonal contrast was most striking for CO and NO₂, both showing sharp and persistent increases each winter and markedly lower levels during warmer months. Given that these combustion-related pollutants rise systematically in cold seasons—coinciding with the winter peak of KD onset counts—they warrant particular attention in the subsequent exposure–response analyses.

### 3.2 Short-term higher CO and NO₂ exposure increases KD risk

**Figure 3:**
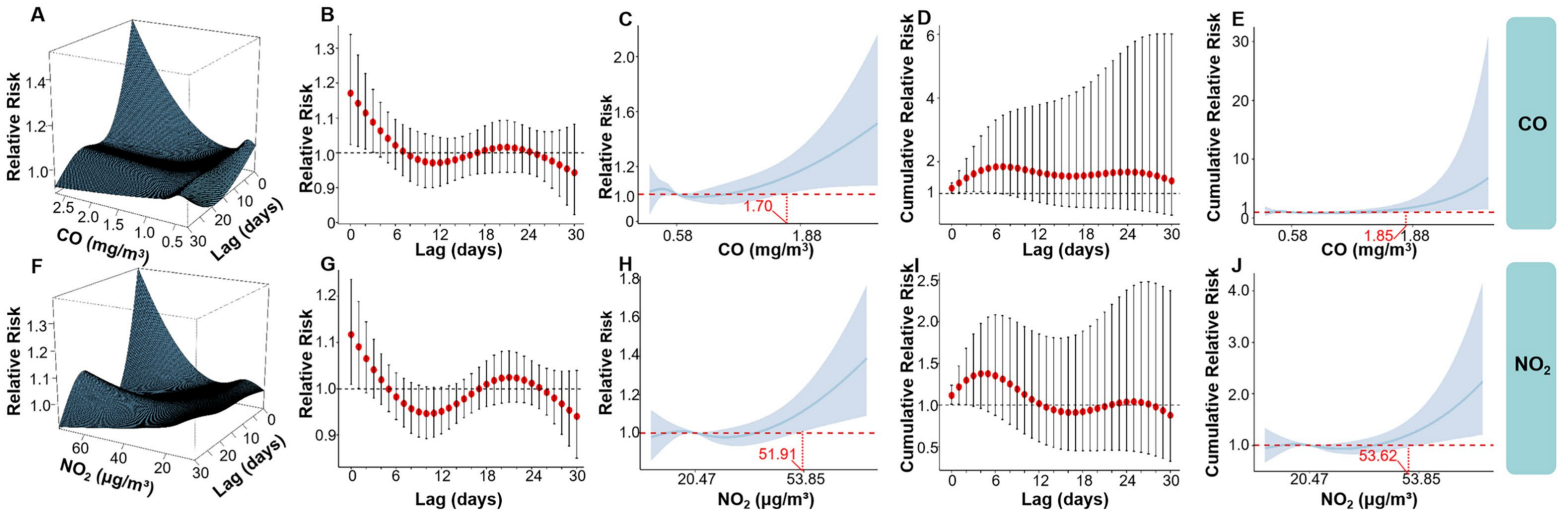
Exposure–lag–response associations of CO and NO₂ with pediatric KD. Panels **(A–E)** depict CO; panels **(F–J)** depict NO₂. Reference concentrations were 0.58 mg/m^3^ for CO and 20.47 μg/m^3^ for NO₂; high-exposure levels were 1.88 mg/m^3^ for CO and 53.85 μg/m^3^ for NO₂. **(A,F)** Three-dimensional exposure–lag–response surfaces showing relative risk (RR) across concentration and lag days. **(B,G)** Lag–response functions comparing high vs. reference exposure. **(C,H)** Exposure–response functions on the day of exposure (lag 0). **(D,I)** Cumulative lag–response functions comparing high vs. reference exposure. **(E)** Cumulative exposure–response for CO over lags 0–6; **(J)** cumulative exposure–response for NO₂ over lags 0–2. Shaded areas indicate 95% confidence intervals; dashed horizontal lines mark RR = 1.

To investigate the effects of combustion-related pollutants on KD onset, we jointly evaluated the lag–response associations for CO and NO₂ using DLNM-based models. When CO exposure was examined using the median concentration (0.58 mg/m^3^) as the reference and the 99.5th percentile (1.88 mg/m^3^) as the high-exposure level, the exposure–lag–response surface (Figure 3A) showed a clear increase in KD risk at higher CO concentrations during the early lag period. Significant single-lag effects were observed at lag 0–3 days, with the strongest effect at lag 0 (RR = 1.172, 95% CI: 1.024–1.340) (Figures 3B,C). Cumulative lag estimates identified lag 0–6 days as the main risk window, yielding a cumulative RR of 1.838 (95% CI: 1.022–3.306) (Figure 3D). The cumulative exposure–response curve indicated a sharp increase in KD risk when CO concentrations exceeded 1.85 mg/m^3^ (Figure 3E).

For NO₂, using the median concentration (20.47 μg/m^3^) as the reference and the 99th percentile (53.85 μg/m^3^) as the high-exposure level, the exposure–lag–response surface similarly demonstrated a short-term increase in KD onset risk (Figure 3F). Significant single-lag associations were detected on lag day 0 (RR = 1.117, 95% CI: 1.009–1.237) and lag day 1 (RR = 1.091, 95% CI: 1.001–1.188), after which the effect gradually diminished (Figures 3G,H). Cumulative lag analysis showed that the most influential exposure window occurred within lag 0–2 days (RR = 1.298, 95% CI: 1.003–1.680) (Figure 3I). The cumulative exposure–response curve further indicated that KD risk increased progressively when NO₂ concentrations exceeded 53.62 μg/m^3^ (Figure 3J).

Taken together, these findings demonstrate that short-term elevations in CO and NO₂—both of which peak during the heating season—are significantly associated with an increased risk of KD onset, highlighting the potential acute triggering role of combustion-related pollutants in susceptible children.

As shown in Supplementary Figure S1, using the median concentrations of O₃, SO₂, PM₂.₅, and PM₁₀ as reference values, we examined the effects of high-concentration of pollutants exposures on KD onset. No statistically significant associations were observed between any of the four pollutants and KD risk within the lag 0–30 day period.

### 3.3 Stratified associations of CO and NO_2_ with KD onset by sex and heating season

**Figure 4:**
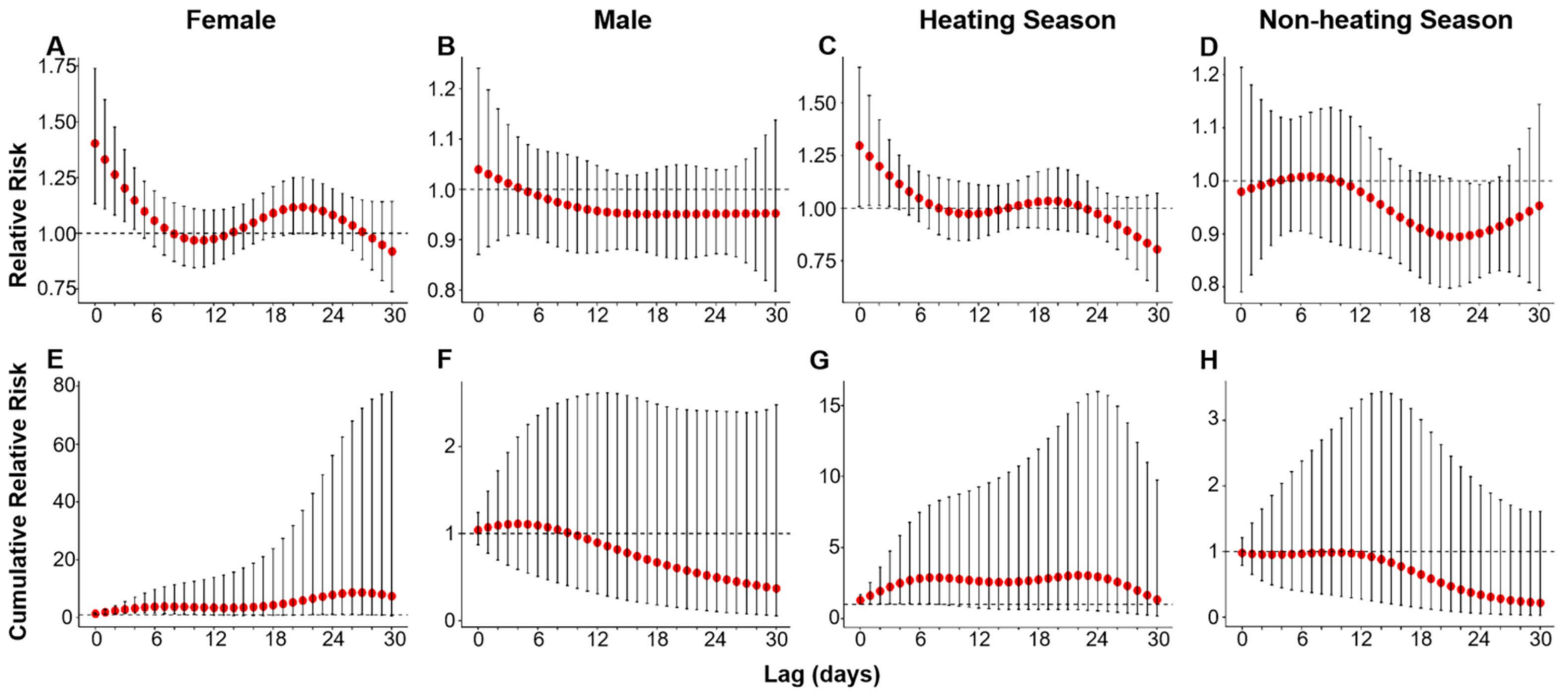
Single-day and cumulative lag effects of high CO exposure on KD onset counts, stratified by sex and heating season. Panels **(A–D)** present the single-day lag–response functions, while Panels **(E–H)** display the corresponding cumulative lag–response functions, with relative risks (RRs) shown on the *y*-axis. Specifically, the sex-stratified associations are shown for females **(A,E)** and males **(B,F)**, and the season-stratified associations are shown for the heating season **(C,G)** and non-heating season **(D,H)**. Shaded regions represent 95% confidence intervals, and the horizontal dashed line indicates the null value (RR = 1).

**Figure 5:**
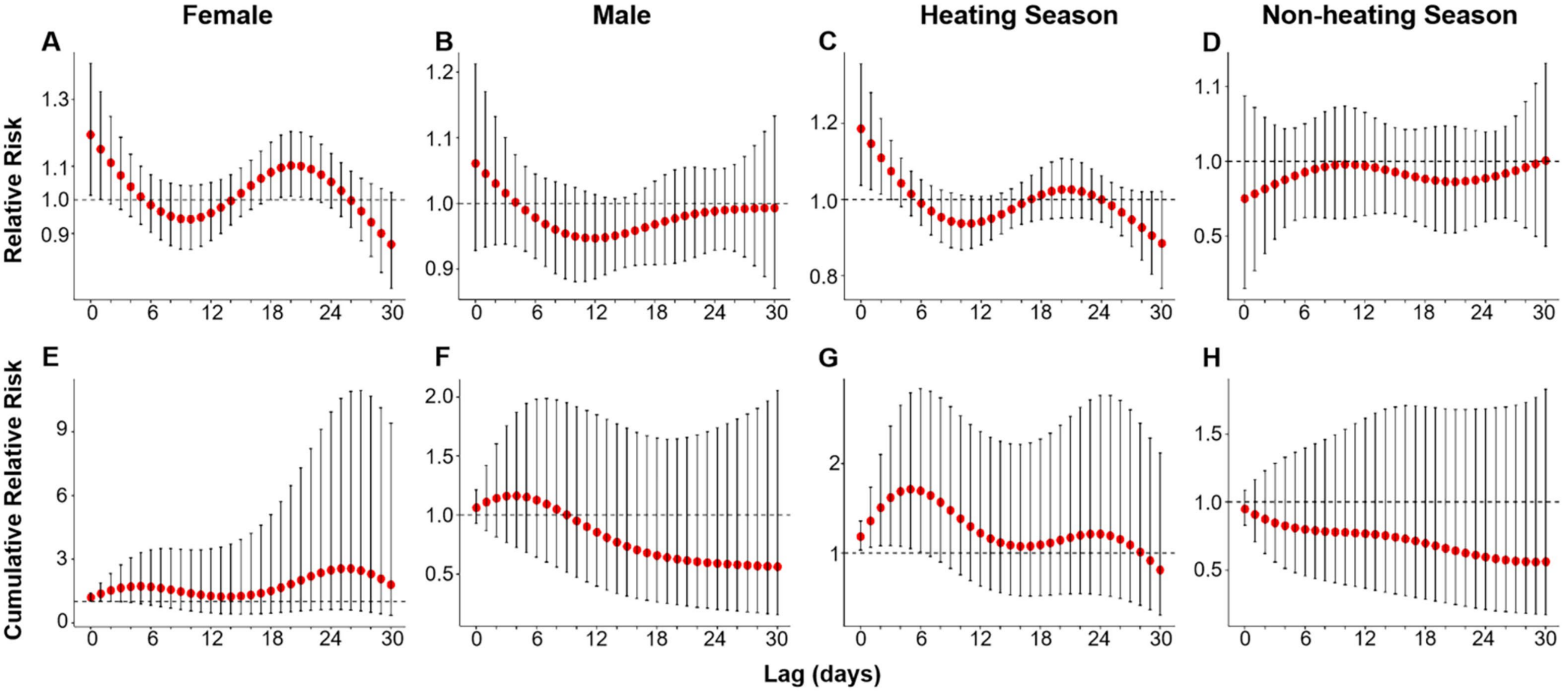
Single-day and cumulative lag effects of high NO_2_ exposure on KD onset counts, stratified by sex and heating season. Panels **(A–D)** present the single-day lag–response functions, while Panels **(E–H)** display the corresponding cumulative lag–response functions, with relative risks (RRs) shown on the y-axis. Specifically, the sex-stratified associations are shown for females **(A,E)** and males **(B,F)**, and the season-stratified associations are shown for the heating season **(C,G)** and non-heating season **(D,H)**. Shaded regions represent 95% confidence intervals, and the horizontal dashed line indicates the null value (RR = 1).

To evaluate whether the associations of CO and NO₂ with KD onset differed by sex or season, we conducted stratified DLNM analyses (Figures 4, 5). For CO, significant short-term effects were observed only in the female subgroup and during the heating season. Among females, elevated CO exposure was associated with an increased KD risk, with the strongest single-lag effects occurring within lag 0–4 days and significant cumulative effects extending across lag 0–7 days (Figures 4A,E). A similar pattern was evident during the heating season, where significant single-lag effects were concentrated within lag 0–3 days and cumulative effects persisted up to lag 0–7 days (Figures 4C,G). In contrast, neither males nor the non-heating season showed statistically significant lag-specific or cumulative associations, with all estimates fluctuating around the null value (RR = 1) (Figures 4B,D,F,H).

For NO₂, the modification pattern was consistent with that observed for CO. In the female subgroup, high NO₂ exposure produced significant short-term effects, with the strongest single-lag risks at lag 0–1 days and cumulative risks remaining significant within lag 0–2 days (Figures 5A,E). Significant associations were also detected in the heating season, where positive effects were concentrated within lag 0–2 days and cumulative effects extended through lag 0–6 days (Figures 5C,G). No significant effects were observed among males or during the non-heating season, where both single-lag and cumulative curves remained close to the null line (Figures 5B,D,F,H).

Furthermore, stratified analyses indicated no evidence of positive associations for the other four pollutants (PM₂.₅, PM₁₀, SO₂, O₃) in any sex or seasonal subgroup across the full 0–30-day lag period (Supplementary Figures S2–S5).

### 3.4 Sensitivity analysis

To assess whether the associations of NO₂ and CO with KD onset were influenced by co-pollutant confounding, two-pollutant DLNMs were constructed using NO₂ and CO as the primary exposures based on the lag periods corresponding to the maximum cumulative risks identified in the single-pollutant models. This approach was used to compare effect estimates under co-adjusted conditions. The effect estimates and their statistical significance for NO₂ and CO remained generally consistent with those observed in the single-pollutant models (Table 2), indicating that their associations with KD were unlikely to be driven by correlated pollutants and demonstrated good independence and robustness. To further evaluate the robustness of the model specification, sensitivity analyses were conducted by varying the degrees of df assigned to key covariates, including long-term temporal trends (6–8 df per year), temperature (4–6 df), and relative humidity (3–5 df). Re-estimation under these alternative df settings did not materially alter the effect estimates (Table 3), supporting the reliability of the findings. Additional sensitivity analyses using more conventional exposure contrasts, including the 75th versus 50th percentile and the 90th versus 50th percentile, showed exposure-response trends generally consistent with the main analysis, although the estimates did not reach statistical significance (Supplementary Figures S7, S7).

**Table 2:** RR with 95%CI for KD onset associated with each pollutant in two-pollutant models.

Pollutant	Adjustment pollutant	RR (95%CI)
CO	NULL	1.838 (1.022–3.306)
	O_3_	1.683 (0.902–3.140)
	PM_10_	2.091 (1.079–4.055)
NO_2_	NULL	1.298 (1.003–1.680)
	O_3_	1.258 (0.959–1.650)
	PM_10_	1.361 (1.021–1.816)

**Table 3:** RR with 95%CI for KD onset associated with each pollutant’s concentration, by different df for calendar time, temperature, and relative humidity.

Variables	Degrees of freedom	CO	NO_2_
Time	6	1.736 (0.982–3.067)	1.253 (0.971–1.617)
	7	1.838 (1.022–3.306)	1.298 (1.003–1.680)
	8	1.920 (1.054–3.495)	1.352 (1.043–1.753)
Temperature	4	1.841 (1.024–3.309)	1.297 (1.003–1.679)
	5	1.834 (1.020–3.298)	1.296 (1.002–1.677)
	6	1.838 (1.022–3.306)	1.298 (1.003–1.680)
Relative humidity	3	1.838 (1.022–3.306)	1.298 (1.003–1.680)
	4	1.805 (1.001–3.255)	1.286 (0.992–1.666)
	5	1.784 (0.988–3.221)	1.293 (0.997–1.676)

## 4 Discussion

This study collected KD hospitalization records from two major medical centers in Heilongjiang Province between 2015 and 2022, and for the first time in a high-latitude cold region, applied a distributed lag non-linear model to systematically evaluate the associations between six major ambient air pollutants and KD onset in children. Unlike previous studies that generally reported peak KD onset counts in summer or summer–autumn seasons (22, 23), we observed in this high-latitude cold region that the daily KD onset counts in winter was comparable to that in summer, suggesting that the seasonal pattern of KD may be influenced by local exposure profiles. An examination of the temporal trends of pollutants indicates that during the heating season, concentrations of combustion-related pollutants—particularly CO and NO₂—exhibited sustained and significant increases and remained at relatively high levels throughout the winter. This discrepancy may be attributable to the accumulation of air pollutants resulting from centralized winter heating, thereby altering the seasonal occurrence pattern of KD.

Regarding the main effects of pollutants, this study found that short-term exposure to higher levels of carbon monoxide (CO, lag 0–3 days) and nitrogen dioxide (NO₂, lag 0–2 days) was significantly associated with an increased risk of KD onset. Notably, the cumulative lag-effect patterns of CO and NO₂ closely corresponded to the seasonal increases in combustion-related pollutants during winter, suggesting that they may act as acute environmental triggers in cold regions. Furthermore, stratified analyses suggested that the observed associations appeared more evident during the heating season and among female children. However, because these subgroup patterns were based on stratified analyses rather than formal interaction tests, they should be regarded as exploratory findings. Direct evidence supporting sex-specific susceptibility to CO- or NO₂-related KD onset remains limited, and future studies with larger sample sizes and formal interaction analyses are needed to evaluate whether sex modifies the association between air pollution and KD onset.

From an exposure perspective, the high-latitude cold region examined in this study experiences markedly elevated concentrations of CO and NO₂ during winter as a result of the combined effects of coal-based centralized heating, traffic emissions, and constrained atmospheric dispersion conditions (24, 25), providing a plausible explanation for the observed “high exposure–acute triggering” pattern. Meanwhile, evidence consistent with our findings remains limited in the existing literature, particularly with respect to NO₂, for which results have been highly heterogeneous. For example, Fujii et al. defined high NO₂ exposure as ≥37.6 μg/m^3^, substantially lower than the observed NO₂ concentration of 53.85 μg/m^3^ applied in the present study (11), and such a lower cutoff may have constrained the detection of significant associations. In addition, many previous studies used hospital admission dates or the timing of intravenous immunoglobulin (IVIG) treatment as proxies for KD onset, which may not accurately capture the true acute onset of the disease. By retrospectively reviewing clinical records to determine the first day of fever, our study was better positioned to detect short-term exposure effects (lag 0–2 days), which may also help explain why prior studies failed to identify the immediate triggering role of NO₂.

The effects of CO and NO₂ observed in this study have clear biological plausibility. Carbon monoxide can bind to hemoglobin to form carboxyhemoglobin, leading to tissue hypoxia, and can induce endothelial injury through oxidative stress and the release of inflammatory mediators (26). Nitrogen dioxide has been shown to increase the generation of mitochondrial reactive oxygen species (ROS), impair myocardial mitochondrial function, and disrupt coronary endothelial function, thereby amplifying inflammatory responses (27). In addition, both pollutants may activate inflammation-related pathways involved in vasculitis, exacerbating inflammatory responses in medium- and small-sized arteries, which closely aligns with the pathophysiological features of KD. Collectively, these mechanisms provide a coherent and compelling biological explanation for the involvement of combustion-related pollutants in the development of KD through multiple immune and vascular processes.

In the present study, short-term exposure to PM₂.₅, PM₁₀, SO₂, and O₃ was not significantly associated with the daily number of KD onsets in the overall or stratified analyses. This finding is broadly consistent with a North American multicity study and a Shanghai time-series study, both of which did not identify stable significant associations between short-term air pollution exposure and KD (28, 29). However, Korean national data and recent meta-analyses have reported associations of PM₂.₅, PM₁₀, or SO₂ with KD in some exposure windows, suggesting potential heterogeneity by region, exposure period, pollutant composition, and model design (12, 30). For O₃, positive associations have been reported in Taiwan (31), East China (32), and Xiamen (33), regions with warmer climates or stronger photochemical activity; in contrast, O₃ in our cold-region study peaked mainly in summer but showed no stable association with daily KD onsets. These findings suggest that the non-significant results for PM₂.₅, PM₁₀, SO₂, and O₃ should be interpreted as reflecting regional heterogeneity rather than absence of biological relevance. In contrast, our findings indicate that in cold-climate regions, CO and NO₂ may serve as indicators of combustion-related pollution associated with KD onset in this cold-region setting, further reinforcing the notion that environmental risk factors for KD exhibit substantial regional heterogeneity.

This study has several limitations. First, consistent with similar studies, daily mean pollutant concentrations from fixed outdoor monitoring stations were used as proxies for population exposure. Although this method provides standardized, long-term, and wide-coverage monitoring data, it cannot capture indoor exposure, individual activity patterns, or spatial heterogeneity, especially in a large area such as Heilongjiang Province where cases were from only two hospitals. This may lead to nondifferential exposure misclassification and affect the accuracy of the findings (34). Second, the latency period of KD remains uncertain, raising the risk of temporal mismatch between exposure and outcome. By retrospectively reviewing medical records, the date of first fever onset was identified as the disease starting point, which is more accurate than using hospital admission dates and may serve as a methodological reference for future studies. Third, due to the lack of reliable daily province-wide surveillance indicators for infectious disease activity, including influenza, enteroviruses, and other respiratory or viral infections, throughout the study period, we were unable to adjust for these factors in the main models. This may have introduced residual confounding; future studies integrating air pollution data with pathogen surveillance or clinical infection indicators are needed to better disentangle the potential contributions of air pollution and infectious triggers to KD onset. Fourth, previous evidence suggests that NO may contribute to KD pathogenesis. However, the absence of concurrent NO monitoring data precluded a comprehensive assessment of its synergistic or interactive effects with NO₂. Fifth, although CO and NO_2_ showed independent stability in the constructed two-pollutant models, the high correlation between them makes it difficult to interpret their pollutant-specific effects. Future multicenter studies combined with mechanistic evidence are still needed to further clarify the promoting effects of individual pollutants on Kawasaki disease. Finally, because time-series analyses are conducted at the population level, they cannot demonstrate individual-level causality. Ambient monitoring data serve only as proxies of personal exposure, and individual susceptibility or behaviors cannot be fully accounted for. Thus, it is not possible to conclude that a specific patient developed the disease directly due to exposure to a particular pollutant.

## 5 Conclusion

In conclusion, this is the first study to demonstrate a significant association between short-term exposure to high CO concentrations and an increased risk of KD among children in high-latitude cold regions of China, while also providing further evidence of a positive relationship between short-term NO₂ exposure and KD risk. In contrast, PM_2.5_, PM_10_, SO_2_, and O_3_ did not show stable significant associations with daily KD onset cases. These findings suggest that regulatory authorities should strengthen the control of CO and NO₂ to mitigate the public health burden of KD in cold regions.

## Data Availability

The raw data supporting the conclusions of this article will be made available by the authors, without undue reservation.
