# Supplementary material for: Short-term exposure to CO and NO2 and Kawasaki disease onset in a cold region of China: a distributed lag non-linear time-series study
Source: Front Public Health. 2026 Jun 17;14:1853103. doi: 10.3389/fpubh.2026.1853103 (PMC13318862; doi:10.3389/fpubh.2026.1853103)
Supplement: Supplementary file 1 [file Data_Sheet_1.docx]

**Supplementary Materials**

**Table**

**Table S1** Spearman Correlation Analysis of PM₂.₅, PM₁₀, SO₂, NO₂, O₃_8h, and CO

**Figures**

**Fig. S1** Lag-specific and cumulative associations of upper-tail O₃, SO₂, PM₂.₅, and PM₁₀ exposure with daily KD onset cases from 2015 to 2022

**Fig. S2** Lag-specific and cumulative associations of upper-tail O₃ exposure with daily KD onset cases, stratified by sex and heating season, from 2015 to 2022

**Fig. S3** Lag-specific and cumulative associations of upper-tail SO₂ exposure with daily KD onset cases, stratified by sex and heating season, from 2015 to 2022

**Fig. S4** Lag-specific and cumulative associations of upper-tail PM₂.₅ exposure with daily KD onset cases, stratified by sex and heating season, from 2015 to 2022

**Fig. S5** Lag-specific and cumulative associations of upper-tail PM₁₀ exposure with daily KD onset cases, stratified by sex and heating season, from 2015 to 2022

**Fig. S6** Sensitivity analysis of single-day and cumulative lag effects of CO and NO_2_ using the 75th versus 50th percentile exposure contrast

**Fig. S7** Sensitivity analysis of single-day and cumulative lag effects of CO and NO_2_ using the 90th versus 50th percentile exposure contrast

**Table S1** Spearman Correlation Analysis of PM₂.₅, PM₁₀, SO₂, NO₂, O₃_8h, and CO

|  | PM_2.5_ | PM_10_ | SO_2_ | NO_2_ | O_3__8h | CO |
| --- | --- | --- | --- | --- | --- | --- |
| PM_2.5_ | 1 | 0.49** | 0.84** | 0.82** | -0.03 | 0.87** |
| PM_10_ | 0.49** | 1 | 0.35** | 0.54** | 0.08* | 0.46** |
| SO_2_ | 0.84** | 0.35** | 1 | 0.78** | -0.15** | 0.83** |
| NO_2_ | 0.82** | 0.54** | 0.78** | 1 | -0.24** | 0.85** |
| O_3__8h | -0.03 | 0.08* | -0.15** | -0.24** | 1 | -0.15** |
| CO | 0.87** | 0.46** | 0.83** | 0.85** | -0.15** | 1 |

Significant correlation at the indicated levels: *P < 0.05, ** P < 0.01.

**Fig. S1.** Lag-specific and cumulative lag effects of O₃, PM₂.₅, PM₁₀, and SO₂ on KD onset. Panels A–D show lag–response relationships for high versus reference pollutant concentrations over lag 0–30 days; panels E–H show the corresponding cumulative lag–response relationships from lag 0 up to each lag day (y-axis: relative risk, RR). Red dots represent point estimates and vertical bars indicate 95% confidence intervals; dashed horizontal lines mark RR=1.

**Fig. S2.** Single-day and cumulative lag effects of high O₃ exposure on KD incidence, stratified by sex and heating season. Panels show lag–response and cumulative lag–response relationships (y-axis: relative risk, RR). Shaded areas indicate 95% confidence intervals; dashed horizontal lines mark RR = 1.

**Fig. S3.** Single-day and cumulative lag effects of high SO₂ exposure on KD incidence, stratified by sex and heating season.

Panels show lag–response and cumulative lag–response relationships (y-axis: relative risk, RR). Shaded areas represent 95% confidence intervals, and dashed horizontal lines indicate RR = 1.

**Fig. S4.** Single-day and cumulative lag effects of high PM₂.₅ exposure on KD incidence, stratified by sex and heating season.

Panels show lag–response and cumulative lag–response relationships (y-axis: relative risk, RR). Shaded areas represent 95% confidence intervals; dashed horizontal lines indicate RR = 1.

**Fig. S5.** Single-day and cumulative lag effects of high PM₁₀ exposure on KD incidence, stratified by sex and heating season.

Panels show lag–response and cumulative lag–response relationships (y-axis: relative risk, RR). Shaded areas represent 95% confidence intervals; dashed horizontal lines indicate RR = 1.

**Fig. S6 Sensitivity analysis of single-day and cumulative lag effects of CO and NO_2_ using the 75th versus 50th percentile exposure contrast**

Panels show the lag–response and cumulative lag–response relationships for CO and NO_2_ using the 75th versus 50th percentile exposure contrast. The y-axis represents relative risk (RR). Shaded areas represent 95% confidence intervals, and dashed horizontal lines indicate RR = 1.

**Fig. S7 Sensitivity analysis of single-day and cumulative lag effects of CO and NO_2_ using the 90th versus 50th percentile exposure contrast**

Panels show the lag–response and cumulative lag–response relationships for CO and NO_2_ using the 90th versus 50th percentile exposure contrast. The y-axis represents relative risk (RR). Shaded areas represent 95% confidence intervals, and dashed horizontal lines indicate RR = 1.
